# BIOLOGICAL SEX MODERATES THE EFFECT OF EXERCISE ON COGNITIVE FUNCTION IN ADULTS WITH CHRONIC STROKE

**DOI:** 10.1093/geroni/igae098.0519

**Published:** 2024-12-31

**Authors:** Rebeca Hernández Gamboa, Ryan Falck, Elizabeth Dao, Teresa Liu-Ambrose

**Affiliations:** University of British Columbia, Vancouver, British Columbia, Canada; University of British Columbia, Vancouver, British Columbia, Canada; University of British Columbia, Vancouver, British Columbia, Canada; University of British Columbia, University of British Columbia, British Columbia, Canada

## Abstract

Exercise (EX) and cognitive/social enrichment (ENRICH) are promising strategies for promoting cognitive function post-stroke. The effects of these interventions on cognitive function may vary by biological sex, i.e. males (M) vs females (F). We conducted a secondary analysis of a three-arm, six-month, assessor-blinded, randomized clinical trial among 120 community-dwelling adults aged 55+ years with chronic stroke (i.e., ≥12 months since stroke), to examine if biological sex moderates the effects of EX or ENRICH on cognitive function. Participants were randomly assigned to 2x/week EX (N=34, M=21, F=13), 2x/week ENRICH (N=34, M=23, F=11), or 2x/week balance and tone control (BAT, M=30, F=22). Our primary outcome of cognitive function was the Alzheimer’s Disease Assessment Scale Cognitive Subscale Plus (ADAS-COG-Plus) at baseline, end of intervention (6 months), and follow-up (12 months). Using linear mixed models, we examined if biological sex moderated the effects of EX or ENRICH on cognitive function using planned contrasts. Baseline ADAS-Cog-Plus and Mini Mental State Exam score were included as fixed-effect covariates. Biological sex moderated the effect of EX, but not ENRICH, on ADAS-Cog-Plus (F = 5.75, p< 0.01). The effects of EX compared to BAT were significantly larger for females than males (estimated mean difference: -0.394, 95% CI: -0.778, -0.011, p< 0.05). EX females had better ADAS-COG-PLUS performance at end of the intervention (estimated mean difference: -0.463, 95% CI: -0.758, -0.168, p< 0.005) compared with females in the BAT group. Findings suggest that exercise may be a key strategy to promote cognitive function among older females living with stroke.

